# Thyroid Cancer in Saudi Arabia: A Histopathological and Outcome Study

**DOI:** 10.1155/2017/8423147

**Published:** 2017-02-27

**Authors:** Ali S. Alzahrani, Haneen Alomar, Nada Alzahrani

**Affiliations:** ^1^King Faisal Specialist Hospital & Research Centre, Department of Medicine, Riyadh, Saudi Arabia; ^2^Department of Molecular Oncology, Riyadh, Saudi Arabia; ^3^Research Centre, Jeddah, Saudi Arabia

## Abstract

Most data on differentiated thyroid cancer (DTC) came from the Western world. We describe its salient characteristics and outcome from a Middle Eastern country. *Patients and Methods*. We studied all cases of TC seen during a 2-year period (2004-2005) seen at our institution. *Results*. A total of 600 consecutive cases of DTC with a median age at diagnosis of 39 years (5–85) and the female : male ratio of 459 : 141 (76.5% : 23.5%). The cases included classical papillary thyroid cancer (PTC) in 77%, follicular variant PTC in 13.3%, follicular thyroid cancer in 3.2%, and other rare subtypes 6.5%. Total or near-total thyroidectomy was performed in 93%, central and/or lateral neck dissection in 64.5% of cases, and radioactive iodine ablation in 82% of cases. Additional therapies were administered to 154 patients (25.7%). At a median follow-up period of 7.63 years (0.22–13.1), 318 patients (53.3%) were in excellent response, 147 (24.5%) having an indeterminate response, 55 (9.2%) biochemically incomplete, 33 (5.5%) structurally incomplete, and 27 (4.5%) unclassifiable. Twenty cases died secondary to DTC (disease-specific mortality 3.3%). *Conclusions*. In Saudi Arabia, DTC is common and occurs at young age and predominantly in females. Although remission is common, persistent disease is also common but disease-specific mortality is low.

## 1. Introduction

Thyroid cancer (TC) is the most common endocrine malignancy. Its incidence has been steadily increasing over the last 4 decades [1–4]. It is now considered the fastest growing human cancer in terms of its incidence [1]. Furthermore, due to the generally favorable outcome of TC, the number of patients on long-term follow-up is high. In the USA, it is expected that 64300 new patients have been diagnosed with TC in 2016 and only 1980 patients (0.3%) have died from it. Similar trends have been observed in many parts of the world [5, 6]. The reasons for this seemingly relentless increase in the incidence of TC are not fully understood, but there is no doubt that the improved diagnostic methods and in particular the widespread use of neck ultrasonography have contributed to this increase in the diagnosed cases of TC [5, 7–9]. Data also suggest a real increase in de novo cases of TC for unclear reasons [10].

Saudi Arabia has witnessed major changes in its population census and economic and cultural structure. Data from the National Cancer Registry have shown a significant increase in the incidence of TC [11, 12]. TC is the second most common cancer in females after breast cancer and its incidence has been also increasing in males [11, 12]. Until recently, most cases were referred to King Faisal Specialist Hospital & Research Center (KFSH & RC) in Riyadh, the premier tertiary care center in Saudi Arabia. The well-maintained and accurate registry at this center also shows a major increase in the annually referred cases of TC [11]. It is the fourth most common treated cancer in general and the second most common in females at KFSH & RC. In this center alone, more than 5000 cases have been treated since the early 1980s and more than 3500 cases are still on long-term follow-up. Obviously, it is important to understand the clinical and pathological features and the management and outcome of this common cancer. There are no recent data on these features of TC in Saudi Arabia. The last study was published in the mid-1990s [13] when the clinical practice, pathological classification, and management tools were significantly different from what is available today. In 1998, we updated our management protocol to be consistent with the international standards of care at that time which recommended total or near-total thyroidectomy for most cases of differentiated TC (DTC) and radioactive iodine (RAI) remnant ablation for the intermediate and high-risk tumors [14, 15]. We have also followed the subsequently released American Thyroid Association (ATA) guidelines that were published in 2006, 2009, and most recently in 2016 [16–18]. Therefore, it is imperative and timely that we review DTC in Saudi Arabia in view of its increasing incidence and the significant changes in its classification and management that took place in the years 1998–2016.

## 2. Patients and Methods

In order to select a representative sample for the period 1998–2016 during which the management practices had become uniform and in accordance with the international standards, we studied all new consecutive cases of TC seen during a 2-year period (1 January 2004–31 December 2005). We choose consecutive cases to avoid selection bias and the years 2004-2005 since they are roughly in the middle of this period and at the same time would allow enough follow-up time to report on the long-term outcomes. An institutional review board was obtained and data were collected from the medical and electronic records and entered in an electronic database (RedCap). The demographic data included age at the time of diagnosis and gender while the histopathological features included tumor size (the largest diameter of a unifocal tumor or the largest focus if multifocal), tumor subtype, multifocality (more than one focus), extrathyroidal extension/invasion, lymphovascular invasion, and lymph node and distant metastasis according to the recommended classification by the ATA guidelines.

In 1998, we updated our management protocol according to the international standards at that time. The diagnosis of patients was established by fine-needle aspiration biopsy under ultrasound guidance. Patients with malignant cytopathology underwent near-total or total thyroidectomy in most cases except those with microcarcinomas in whom unilateral hemithyroidectomy is usually performed. Patients who are referred to our center with partial thyroidectomy would routinely undergo completion thyroidectomy if they have PTC > 1.5 cm, widely invasive FTC, or minimally invasive FTC > 4 cm. Prophylactic central neck dissection was performed for stage III and IV tumors. Therapeutic central ± lateral modified neck dissection was performed when pathologically looking lymph nodes were seen preoperatively on ultrasonography or intraoperatively by direct examination. Following surgery, patients were assessed for their need of radioactive iodine (RAI) remnant ablation or therapy. Thyroid remnant ablation was routinely performed for patients with PTC > 1.5 cm and for patients with widely invasive FTC and any type of tumor if diagnostic radioiodine whole-body scans showed an uptake outside the thyroid bed. The administered activity used to be 100 mCi for remnant ablation, 100–150 mCi if there are lymph node metastases, and 150–200 mCi if there are distant metastases. In the last 5 years, a trend towards more conservative doses of RAI was adopted with most patients receiving 30–100 mCi for thyroid remnant ablation and 100–200 mCi for patients with lymph node or distant metastases. In the vast majority of cases, RAI ablation/therapy was performed using thyroid hormone withdrawal rather than recombinant human TSH stimulation. Thyroid hormone suppression was prescribed to achieve TSH suppression < 0.001 for the vast majority of cases although a risk-based TSH suppression level has been adopted in the last 5 years. Patients were assessed every 6–12 months by stimulated or unstimulated Tg and Tg autoantibodies, TSH, FT4, and neck ultrasonography. DxWBS were sometimes performed for high-risk patients and patients suspected to have residual or distant disease. Other imaging studies including merged F-18-fluorodeoxyglucose positron emission tomography-computed tomography (PET-CT), CT scans, MRI, and others were sometimes used as needed.

We assessed the outcome at 6–12 months after RAI ablation or primary surgery (if RAI ablation was not done). We used the ATA response to therapy system to assess the outcome of patients [19]. When suppressed Tg level is <0.2 ng/mL or stimulated Tg < 1 ng/mL in the absence of Tg autoantibodies and negative imaging studies, patients were labelled to have achieved an excellent response to therapy. Patients with suppressed Tg of 0.2–1 ng/dl or stimulated Tg 1–10 ng/dl, or positive Tg autoantibodies and/or nonspecific ultrasonographic findings, or faint uptake in the thyroid bed on DxWBS were labelled to have an indeterminate response. Suppressed Tg ≥ 1 ng/dl or stimulated Tg ≥ 10 ng/dl (with negative Tg autoantibodies) or rising Tg autoantibodies in the absence of clear foci of disease were considered biochemically incomplete. Finally, when the disease is anatomically or cytopathologically confirmed, patients were considered to have structurally incomplete response [19].

### 2.1. Statistical Analysis

Continuous variables are expressed as mean ± SD or median and range and a *t*-test was used for comparison. Categorical data are expressed as numbers and percentages and chi square or Fisher's exact tests were used for comparison and association analyses. A multivariate logistic regression analysis was used to analyze predictors of outcome. In all comparisons, a two-tailed *P* value < 0.05 was considered significant.

## 3. Results

### 3.1. Demographic Data

**Figure 1:**
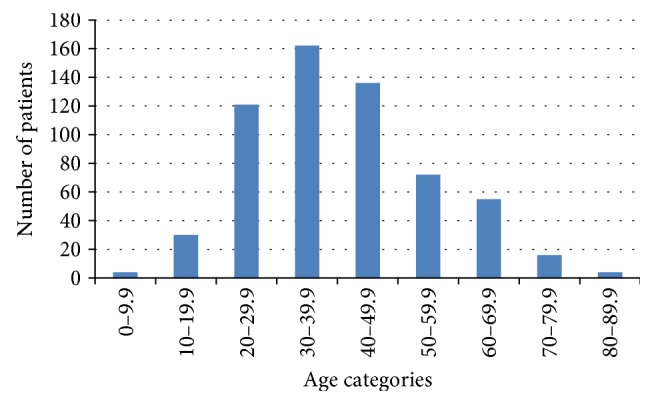
Age distribution of 600 cases of differentiated thyroid cancer seen during the period 2004-2005.

The median age at diagnosis was 39 years (5–85). The majority occurred in the fourth and fifth decades of life (interquartile range 29–49 years, Figure 1). There were 459 (76.5%) females and 141 (23.5%) males with female to male ratio of almost 3 : 1 (Table 1). The mean age at diagnosis was significantly older in males (45 ± 16.6 yrs) compared to females (38.6 ± 14.4 yrs) (*P* < 0.0001).

### 3.2. Histopathological Features

Of 629 total cases of thyroid cancer seen during the period 1 January 2004–31 December 2005, 14 (2.2%) were MTC and 15 (2.4%) were ATC. These subtypes of TC are not included in further analysis. The remaining 600 follicular cell-derived TC and their subtypes are shown in Table 2. The vast majority (77%) are of the classical papillary thyroid cancer (CPTC) followed by the FVPTC subtype (13.3%). Other subtypes are rare including FTC which occurred only in 3.2%. Unexpectedly, the median tumor size was small (2.15 cm) indicating that our patients' tumors are similar in size to those of other populations and suggesting that the impact of widespread use of imaging contributed to detection of thyroid cancer at an early stage and the apparently increasing incidence in our population. Tumor multifocality, extrathyroidal tumor extension/invasion, and lymphovascular invasion were common (Table 1). Lymph node metastases occurred in about 60% and distant metastasis occurred in 13.3% (Table 1). Interestingly, about 60% of cases had Hashimoto's thyroiditis or nodular goiter in the background thyroid tissue. About 70% of cases were in TNM stage I while the rest were evenly distributed between stages II, III, and IV (Table 1).

### 3.3. Management

Near-total or total thyroidectomy was performed in 93% of cases. This was achieved in 1 surgery in 351 patients (58.5%), 2 surgeries in 198 patients (33%), 3 surgeries in 29 cases (4.8%), and 4 surgeries in 5 patients (0.8%). Surgery was not done in 17 cases due to either significant comorbidities, very old age, and poor prognosis or no return for therapy. Lymph node dissection was performed in 277 cases (46.2%). In 33 cases (5.5%), this was limited to the central compartment while it included central and unilateral or bilateral compartment dissection in 220 cases (36.7%). In 24 cases (4%), the site of dissection was unclear. RAI was given to 492 cases (82%) for either remnant ablation or therapy of local or distant metastases. The median administered activity was 147 mCi (range 19–302).

### 3.4. Short-Term Outcome

At 6–12 months after thyroid surgery and the first RAI ablation/therapy, 269 patients (44.8%) achieved the ATA excellent response status, 195 (32.5%) patients had biochemically incomplete (6%) or structurally incomplete (26.5%) status, 76 (12.7%) patients had an indeterminate status, and one patient died due to progressive metastatic poorly differentiated thyroid cancer (Table 3).

## 4. Additional Therapeutic Interventions

In 446 (74.3%), no further therapeutic intervention was performed. In the other 154 cases, one or more therapeutic interventions were performed (Table 3). Another single surgery was undertaken in 45 cases (7.5%), one additional dose of RAI in 44 cases (7.3%), and more than one intervention in 65 cases (10.8%).

## 5. Final Outcome

As shown in Table 3, at a median follow-up period of 7.63 years (0.22–13.1), 318 patients (53.3%) achieved an excellent response and 147 (24.5) were in an indeterminate status while 88 patients had either a biochemically incomplete (9.2%) or structurally incomplete (5.5%) status. Twenty patients died secondary to thyroid cancer (disease-specific mortality 3.3%).

## 6. Predictors of Short- and Long-Term Outcome

As shown in Table 4, in a univariate analysis, male gender, tumor extrathyroidal invasion, lymphovascular invasion, and lymph node and distant metastasis predicted persistent disease in the short-term follow-up (6–12 months after initial management). In the long term, male gender, age at diagnosis, extrathyroidal extension/invasion, lymphovascular invasion, and lymph node and distant metastasis predicted the presence of disease (Table 5).

In a multivariate logistic regression analysis in which male gender, age at diagnosis, extrathyroidal extension, lymphovascular invasion, and lymph node and distant metastases were entered as potential predictive factors of disease (biochemically or structurally incomplete status or death), only age at diagnosis and extrathyroidal extension remain significant predictors of having a disease at the last follow-up (Table 5).

## 7. Discussion

The incidence of DTC has been steadily increasing in many parts of the world. Saudi Arabia is not an exception. A recent report that reviewed the epidemiological aspects of all DTC cases seen during the period 2000–2010 at KFSH & RC showed a significantly steadily increasing incidence between 2000 and 2010 [11]. Indeed, the general demographic and epidemiological data in that report are similar to our study assuring us that our sample is quite representative of the DTC in our hospital and in the whole country since the vast majority of patients are referred to KFSH & RC. In that epidemiological report, a total of 2292 cases of TC were seen during the 10-year period. It showed a gradual and steady increase in the number of new cases over this period. Our study included about one-third of these cases (629 cases). Gender (76.3% females), age distribution (median age 38 years), histopathological subtypes, and stage of the cancer are all similar to our findings. All of these features are additional assurances of the representativeness of our study.

In the current study, we have characterized the salient features of DTC in Saudi Arabia by reviewing the clinical, pathological, and therapeutic features and outcome of a large sample of TC seen over a carefully selected representative 2-year period. The study shows that DTC is a disease of the young to the middle-age with a median age at the time of diagnosis of 39 years and the majority of cases occurring in the fourth and fifth decades. The interquartile range is 29–49 years and 65% of our patients are <45 years of age which is taken as a cutoff age limit in the AJCC risk of mortality classification. Indeed, similar median ranges were reported before in a study from the same institution with the median age in men of 48 years and in women of 35 years [13]. More recently, another study that included all patients seen during the period 2000–2010 reported a median age of 38 years and the highest incidence in the age range 30–39 years [11]. These numbers are significantly lower than those reported from SEERS data where the median age at diagnosis is 51 years and most cases occur in the age range of 45–54 years (Cancer of The Thyroid-SEER Stat Fact Sheets, available at https://seer.cancer.gov/statfacts/html/thyro.html accessed on June 27, 2016). Similar to previous studies, DTC is much more common in females than males with a ratio of about 3 : 1.

ATC was diagnosed in 2.5% of cases. Although low, this rate is higher than the generally reported international rates of about 1-2% [20, 21]. FTC occurred only in 4.5% in this cohort which is lower than the generally reported international incidence of around 10% [22] but remained similar to the rates reported in previous studies from the same institution [23]. In a previous study from our institution covering 3 years and including 246 cases of TC, ATC occurred in 3 cases (1.25) and FTC occurred similarly in only 3 cases (1.2%) [13]. Similar to the previous studies from our institution and others, PTC and its subtypes dominated the cases of TC. The majority was of the classical subtype, but the follicular variant PTC was also common occurring in 13.3% of cases. This variant was not well recognized before the 2000s and was frequently referred to as FTC or mixed FTC and PTC [13, 24, 25]. This may explain the seemingly increasing rate of this variant in the current study compared to previous studies. The median tumor size in our patients was 2.15 cm which is not different from many other international studies. However, it is significantly lower than that reported in an earlier study from the same institution (5 cm in men and 4 cm in women) [13] suggesting that, with improved method of detection and more widespread use of neck ultrasonography, we are detecting tumors at smaller tumor size than in the past. There was a high rate of extrathyroidal invasion, tumor multifocality, lymphovascular invasion, and lymph node and distant metastases and that may explain the relatively low rates of disease-free status in the short-term and at the final outcome. Most of these factors predicted the persistent disease at 6–12 months after the initial management and the persistent/recurrent disease at the last follow-up visit (Tables 4 and 5). These findings are similar to previous studies and indicate that tumors with these features are more aggressive and less likely to be eradicated. Patients who achieved remission rarely developed recurrence. This is consistent with the relatively recently introduced concept of dynamic staging that was initially proposed by Tuttle et al. and adopted in the last ATA guidelines [18, 26–28]. This concept emphasizes the fact that staging of DTC varies over time such that what might be staged as a high-risk tumor initially may change to low or intermediate risk after therapy and vice versa [27, 28]. Indeed, more than a quarter of our patients needed one or more additional therapeutic interventions due to persistent biochemical or structural disease. The cancer-specific mortality was low but higher than those reported in other studies. Only 20 out of a total of 600 cases (3.3%) died over a median follow-up period of 7.63 years secondary to DTC.

Although we have not specifically studied the mutational profile of our patients in this study, we have recently reported on the rates of *BRAF*^V600E^ and *TERT* promotor mutations in our patients and their associations with several aggressive histopathological features and outcome [29, 30]. *BRAF*^V600E^ occurs in about 46.5% of our patients and is associated with age at diagnosis, extrathyroidal invasion, lymph node metastases, and higher TNM stage [29]. *TERT* promotor mutations occurred in about 13% of all cases with increasing rates in the higher stage disease and poorly differentiated subtypes. It was associated with increasing age, tumor size, extrathyroidal invasion, vascular invasion, higher TNM stage, *BRAF*^V600E^ mutation, and persistent/recurrent disease [30].

Two previous studies were published from the same institution of the current series in the mid-1990s. al-Nuaim et al. reported in 1996 on 233 cases of PTC, and the majority (88%) was of the classical subtype and 7% were <1.5 cm [13]. In that study, >80% of the tumors were >2 cm and about 50% of these were >4 cm [13]. By contrast, the current study shows that 20% of cases are microcarcinomas (≤1 cm), about 50% of tumors are ≤2 cm, and about 75% of cases are ≤3.5 cm. This suggests that more cases with small size tumors including microcarcinomas are being detected, most probably due to wider use of imaging studies, particularly ultrasonography of the neck. Similar to the current study, the median age was 37 years and men were significantly older (median age 48 years) at the time of diagnosis than women (median age 35 years) [13].

Ahmed et al. reported on 875 cases of TC seen at KFSH & RC between 1975 and 1989 [23]. PTC occurred in 79% of cases and ATC, MTC, FTC, thyroid lymphoma, and Hurthle cell carcinomas occurred in 5.4%, 5.3%, 4.3%, 3.6%, and 0.9%, respectively [23]. These percentages contrast with significantly different percentages in the current study reflecting a possible change in thyroid histopathological pattern and/or improvement and change in thyroid histopathological classification. The mean age at the diagnosis for males was 50 years and that for females was significantly younger at 42 years of age [23]. TC occurred almost equally during the third, fourth, and fifth decades of life, but there were differences when gender was considered with most cases of females occurring in the third decade (20–30 years) and most cases of thyroid cancer in males occurring in the fifth decade (40–50 years) [23]. This contrasts with the current study where the median age at diagnosis is 39 years and the interquartile range is 29–49 years. The mean ages for females and males are about 5 years younger than those reported in Ahmed et al.'s study (45 for males and 38.6 for females). This suggests an early detection of cases, better health awareness, or a true change in the pattern of the disease with a younger age of onset compared to the past.

Our study has a number of strengths and limitations. It included a large sample from the main tertiary care center in Saudi Arabia where the practice and management of TC have been standardized for a long time according to the international standards. Although these are hospital data, they are essentially representative of TC in the whole country considering that at the time of the study, almost all patients newly diagnosed with TC are referred to KFSH & RC. It has fairly long-term outcome data which have not been reported before from Saudi Arabia. On the other hand, the data are retrospective and not all data were available on all patients. However, this is the nature of any retrospective study and fortunately the number of patients with deficient data in any parameter is small.

## Figures and Tables

**Table 1:** Characteristics of 600 cases of follicular cell-derived thyroid cancer seen during the period 2004-2005.

Characteristic	Number (%)
Median age (range) years	39 (5–85)
Gender (female : male)	141 : 459 (23.5% : 76.5%)
Tumor size (cm)^∗^	2.15 (0.1–13)
Tumor multifocality^∗^	241/469 (51.4%)
Extrathyroidal extension^∗^	209/495 (42.2%)
Lymphovascular invasion^∗^	70/190 (36.8%)
Hashimoto's or nodular goiter^∗^	343/600 (57.2%)
Lymph node metastasis^∗^	277/465 (59.6%)
Distant metastasis^∗^	77/580 (13.3%)

TNM stage^∗^	
I	384/553 (69.4%)
II	49/553 (8.9%)
III	61/553 (11%)
IVA	14/553 (2.53%)
IVB	3/553 (0.54%)
IVC	42/553 (7.6%)

^∗^Numbers represent number of patients with the characteristic divided by the number of patients with available data. Data were not always available for every patient. Data for tumor size were available for 512 patients, for tumor multifocality in 469 patients, for extrathyroidal extension in 495 patients, for lymphovascular invasion in 190 cases, for lymph node metastasis in 465, for distant metastasis in 580 cases, and for tumor TNM staging in 553 patients.

**Table 2:** The distribution of follicular cell-derived thyroid cancer cases (excluding 15 ATC and 14 MTC) seen during the period 2004-2005.

Tumor type	Number (%)
Classical papillary thyroid cancer	462 (77%)
Follicular variant papillary thyroid cancer	80 (13.3%)
Tall-cell papillary thyroid cancer	16 (2.7%)
Diffuse sclerosing papillary thyroid cancer	8 (1.3%)
Follicular thyroid cancer, widely invasive	9 (1.5%)
Follicular thyroid cancer, minimally invasive	10 (1.7%)
Hurthle cell cancer	8 (1.3%)
Others	7 (1.2%)
*Total*	*600 (100%)*

**Table 3:** Short- and long-term outcome of 600 DTC patients treated during the period 2004-2005.

*Outcome at 6–12 months after RAI*	
Excellent response Indeterminate Biochemically incomplete Structurally incomplete Unclear Death	269 (44.8%) 76 (12.7%) 36 (6%) 159 (26.5%) 59 (9.8%) 1 (0.2)

*Additional therapeutic interventions*	
None One surgery alone One RAI alone More than one interventions	446 (74.3%) 45 (7.5%) 44 (7.3%) 65 (10.8%)

*Outcome at the last follow-up visit*	
Excellent response Indeterminate Biochemically incomplete Structurally incomplete Death Unclear	318 (53.3%) 147 (24.5%) 55 (9.2%) 33 (5.5%) 20 (3.3%) 27 (4.5%)

**Table 4:** Univariate analysis of predictive factors of outcome in the short term (6–12 months after RAI ablation).

Outcome at 6–12 months after RAI			
Characteristic	Remission	Persistent disease	*P* value
Male gender	41/98 (41.8%)	57/98 (58.2%)	<0.0001
Age (yrs)	37.9 ± 12.7	40.4 ± 16.3	0.067
Tumor size (cm)	3.4 ± 2.6	3.6 ± 2.3	0.74
Tumor multifocality	66/189 (44.6%)	82/191 (55.4%)	0.11
Extrathyroidal extension	68/220 (30.9%)	98/159 (61.6%)	<0.0001
Lymphovascular invasion	20/91 (22%)	36/61 (59%)	<0.0001
Lymph node metastasis	98/265 (35.8%)	133/194 (68.6%)	<0.0001
Distant metastasis	17/266 (6.4%)	44/194 (22.7%)	<0.0001

**Table 5:** Univariate and multivariate analyses of demographic and histopathological predictive factors for the outcome at the last follow-up visit.

Outcome at final follow-up					
Unadjusted				Adjusted	
Characteristic	Remission	Persistent/recurrent disease	*P* value	Odds ratio (95% CI)	*P* value
Male gender	52/318 (16.4%)	80/225 (31.4%)	<0.0001	1.48 (0.64–3.4)	0.36
Age at diagnosis (yrs)	36.7 ± 12.9	43.5 ± 15.6	<0.0001	1.039 (1.012–1.07)	0.005
Tumor size (cm)	3.3 ± 2.3	3.4 ± 2.4	0.84		
Tumor multifocality	143/272 (52.6%)	92/186 (49.5%)	0.58		
Extrathyroidal extension	85/258 (32.9%)	115/199 (57.8%)	<0001	3.26 (1.43–7.42)	0.005
Lymphovascular invasion	29/110 (26.4%)	39/77 (50.6%)	0.001	1.52 (0.69–3.34)	0.30
Lymph node metastasis	128/313 (40.9%)	143/248 (57.7%)	<0.0001	1.11 (0.51–2.43)	0.79
Distant metastasis	19/316 (6%)	55/248 (22.2%)	<0.0001	2.11 (0.77–5.78)	0.15
